# Transcranial Laser Stimulation Research—A New Helmet and First Data from Near Infrared Spectroscopy

**DOI:** 10.3390/medicines5030097

**Published:** 2018-09-03

**Authors:** Gerhard Litscher

**Affiliations:** Research Unit for Complementary and Integrative Laser Medicine, Research Unit of Biomedical Engineering in Anesthesia and Intensive Care Medicine, TCM Research Center Graz, Medical University of Graz, Auenbruggerplatz 39, EG19, 8036 Graz, Austria; gerhard.litscher@medunigraz.at; Tel.: +43-316-385-83907; Fax: +43-316-385-595-83907

**Keywords:** transcranial laser stimulation, laser therapy, wavelength, stroke, dementia, mental diseases, near infrared spectroscopy, laser helmet

## Abstract

This editorial adopts a future-oriented technology. It describes a new modern helmet for transcranial laser stimulation and a way to quantify effects of this possible therapeutical method using near-infrared spectroscopy.

Transcranial laser stimulation has become increasingly important, especially in recent times. This is owing to the fact that in the next few years there will be an enormous worldwide increase in so-called mental diseases such as stroke, dementia, Alzheimer’s, or Parkinson’s. Since the conventional therapy successes are rather low, one looks for new medical strategies. Such a method could be transcranial laser stimulation. Initial successes have already been scientifically proven, yet there is currently a lack of useful devices for therapeutic procedures [1,2,3]. Weber Medical (Lauenförde, Germany) has developed a prototype of such an innovative device. At the TCM Research Center (chairman: Gerhard Litscher) of the Medical University of Graz the first promising test measurements were carried out with this helmet, see Figure 1. The first data of this pilot measurement are presented here as part of this editorial.

The prototype system is currently based on infrared lasers using a wavelength of 810 nm. This wavelength has recently been proven (July 2018) to be one of the best for transcranial laser stimulation [4,5]. The authors employed Monte Carlo modeling and a visible human phantom to compute the penetrated photon fluence distribution within the cerebral cortex. By comparing the fluence distribution, penetration depth, and the intensity of the laser-tissue interaction they found that 810 and 660 nm performed much better than other wavelengths [5]. This confirms earlier results from our research group [1,2,3,4].

For the new helmet, altogether 360 infrared lasers with different wavelengths can be used, see Figure 1. The current investigation was performed with 32 active lasers using the parameters indicated in Figure 1. The duration of the stimulation was 20 min.

For the measurement of the changes of the regional oxygen saturation (rSO_2_), an INVOS 5100C Oximeter (Somanetics Corp., Troy, MI, USA) was used. The principle of this system is based on NIRS (near-infrared spectroscopy) technology, which is a noninvasive method for measuring regional oxygenation through the intact skull and has been applied successfully in research and numerous clinical indications for many years [6]. Near-infrared light (730 and 805 nm) is emitted through the skin and after passing through different kinds of tissue (skin and bone) the returned light is detected at two distances from the light source (3 and 4 cm). Based upon this principle, the spectral absorption of blood in deeper structures (2–4 cm) can be determined and defined as rSO_2_. Before starting the measurement, the skin was cleaned with the enclosed skin-prep pad. Then, two sensors were applied in the frontal area on the right and left side of the brain of a healthy volunteer, see Figure 2. To minimize the influence of external light, the head in this area was covered with an elastic band during the recording and stimulation procedure. After a resting time of 15 min, the laser stimulation started. The results of the three phases ((a) before, (b) during, and (c) after stimulation) are indicated in Figure 2. Note the increase in rSO_2_ (left and right) during and after transcranial laser stimulation.

The research on transcranial laser therapy is very interesting and fast-paced. The results are very promising however further research work must certainly be invested in order to be able to use this new helmet as a therapeutic method, for example.

## Figures and Tables

**Figure 1:**
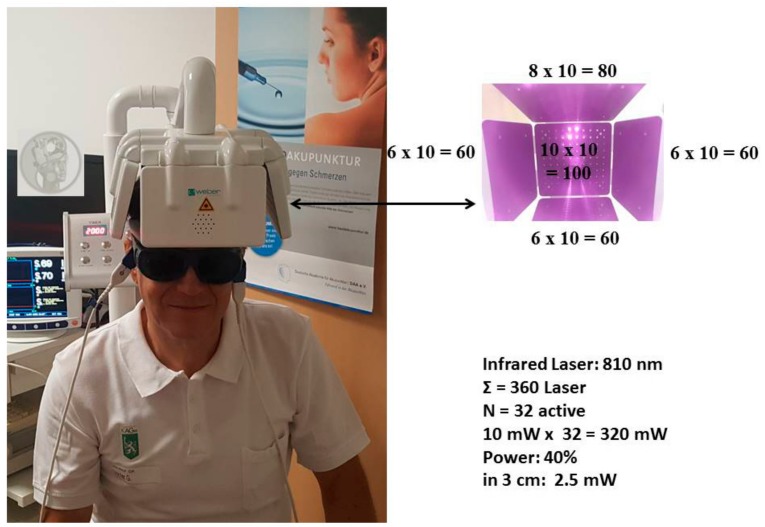
First measurement with the new transcranial laser stimulation helmet from Weber Medical at the TCM Research Center at the Medical University of Graz performed on the 27 August 2018.

**Figure 2:**
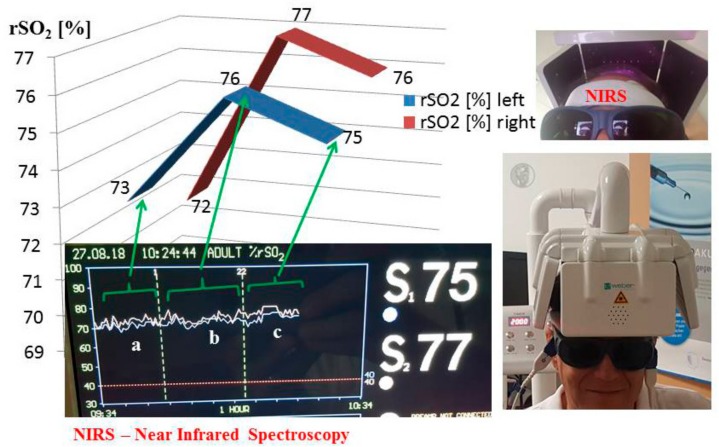
Results of the first pilot measurement of the new transcranial laser stimulation helmet.
